# First-time mothers’ experiences of receiving proactive telephone-based peer support for breastfeeding in Australia: a qualitative study

**DOI:** 10.1186/s13006-022-00476-7

**Published:** 2022-04-22

**Authors:** Fiona E. McLardie-Hore, Della A. Forster, Touran Shafiei, Helen L. McLachlan

**Affiliations:** 1 Judith Lumley Centre, La Trobe University, Melbourne, VIC Australia; 2 Royal Women’s Hospital, Melbourne, VIC Australia; 3 School of Nursing and Midwifery, La Trobe University, Bundoora, VIC Australia

## Abstract

**Background:**

The RUBY randomised controlled trial was found to be effective in promoting breastfeeding continuation, in the setting of a high income country, through a program of proactive telephone-based peer support in the first 6 months postpartum. This paper explores women’s experiences of receiving the peer support intervention in the RUBY trial.

**Methods:**

Ten in-depth, face-to-face interviews were conducted between December 2015 and November 2016 in Metropolitan Melbourne, and regional Victoria, Australia. Participants were women who received the peer support intervention in the RUBY trial and were between 11 and 15 months postpartum at the time of interview. Interviews were underpinned by social support theories and were analysed using inductive thematic analysis.

**Results:**

A global theme of ‘non-judgemental support and guidance’ was identified, which included five organising themes. Four of the organising themes centred on the support from the peer, in which women felt the support was a ‘positive experience with empathy and understanding’, ‘non-judgemental’, ‘practical advice’, and a ‘social connection that was more than just breastfeeding’. In contrast to the support from peers was the theme ‘not all support from family and friends is supportive’.

**Conclusion:**

Participants, including those who considered that they had adequate and available family and friend support for breastfeeding, valued and appreciated the non-judgemental, empathetic and understanding support from peers. This support, facilitated by the anonymity of the telephone-based program, allowed open and honest conversations, normalising women’s experiences and helping them feel less alone in their challenges with breastfeeding and transition to motherhood. These findings can inform the design, and upscaling, of innovative and sustainable peer support models, ensuring delivery of effective and engaging support with a broad population reach.

## Background

Peer support has become increasingly used by communities, families and individuals as a method to manage a range of physical [1, 2] and mental health [3] issues, as well as helping to manage and cope with illness [4, 5] and disability [6], through health promotion, health maintenance, and disease prevention. This support can be derived from family and friends, or more formally structured through community groups or created networks, and may build on professional support [7] or, alternatively, fill a shortfall in care [8]. Peer support is described by Dennis as encompassing the “provision of emotional, appraisal, and informational assistance by a created social network member who possesses experiential knowledge of a specific behaviour” [9]. These models of support-enhancing interventions are recognised for their ability to reach minority and disadvantaged groups, [10] and are emerging in many countries as standard practice. The World Health Organization (WHO) promotes peer support models of care, asserting that through training and guidance, communities can build ‘the knowledge and skills to implement person-centred’ approaches [11]. Globally, health care systems are experiencing increasing operational and economic burdens [12] and must ensure resources are used most effectively, whilst continuing to provide high quality care. Innovative and sustainable approaches are needed to address these resource shortages, with peer support seen as one of the most promising approaches to health care, with reach and coverage for countless populations [13].

There is evidence that breastfeeding is one public health measure that can benefit from peer support programs [14]. Public health recommendations from the WHO are that babies should be exclusively breastfeed until six-months of age, setting global nutrition goals of 50% exclusive breastfeeding by 2025 [15]. Australia’s National Breastfeeding Strategy [16] has adopted these goals, which would see national exclusive breastfeeding at six-months of age rise from a baseline of approximately 15%, [17] to 40% by 2022, and an overall rise of 35% to achieve the 50% target by 2025. Strategies outlined to achieve these national figures include investing in services provided by peer supporters, strengthening existing programs that provide mother-to-mother support and peer counselling, and proactively offering this support to women who wish to breastfeed [16]. With breastfeeding seen as a health priority, peer support has the potential to help achieve these population targets, yet few programs have realised this potential.

The 2017 Cochrane review on ‘Support for healthy breastfeeding mothers with healthy term babies’ [18] found that peer support interventions conducted in low- or middle-income countries had proven effective on any and exclusive breastfeeding maintenance, yet found little evidence in high-income countries. Models varied in method and timing of delivery, number of contacts, and duration of support. Some programs were reactive in nature, relying on women to make contact when they felt they needed help, while others offered proactive contact. Three studies included in this review were telephone-based support, however only one was associated with increased breastfeeding duration (measured at 3 months)[14]. Since this review, a 2019 Australian randomised controlled trial (RCT), Ringing up about breastfeeding early (RUBY), conducted from 2013 to 2016, found a positive association with providing proactive telephone-based peer support and breastfeeding maintenance to six months postpartum [19], in the context of a high-income country.

The RUBY trial offered proactive telephone-based peer support to breastfeeding, first time mothers, in conjunction with standard supports available in hospital, and the community, and tested if this increased breastfeeding maintenance rates at 6 months compared to women with access to only standard supports[20]. Volunteer mothers who had personal breastfeeding experience for at least six months and attended four hours of peer volunteer training provided the peer support in the RUBY RCT. Through empathy, active listening, sharing experiential knowledge and referring mothers to community supports and services as needed, the aim was that the peers helped the mothers reach their intended breastfeeding goals. At recruitment, mothers were advised to expect the first telephone call from their peer in the first few days after giving birth and being discharged from hospital, and that a second call would follow a few days later, then calls would be weekly for the first 12 weeks, then every three or four weeks until six months, and that the mothers could call their peer in between these contacts as needed [19]. This schedule was chosen to reflect that more support was likely to be needed in the first 12 weeks, but the suggested schedule was flexible, and the mothers and peer volunteers could adjust it as needed or desired. Peer volunteers were provided with the mothers first name, phone number, baby’s date of birth (to ensure appropriate timing of the first call) and the baby’s sex. No other information about the mother was provided to the peer.

With little evidence of effective peer support programs in high income settings, it is vital to gain a greater understanding of what elements of the support helped women in the RUBY trial. A cross-sectional survey of women receiving the intervention in the RUBY study found most women reported the support to be helpful, convenient and accessible. Women described the continuity of the peer (that is having the same peer providing support) important and that the peer provided practical and realistic advice and was understanding of what the mother was experiencing [21]. Qualitative studies of women’s experience of breastfeeding peer support have reported that the peer support was valued [22, 23], and promoted positive attitudes toward [24] and improved knowledge of breastfeeding [24, 25]. It has also been reported that support from a peer has helped normalise the mothers’ experiences [26, 27], provided reassurance and helped women cope with the challenges [28], however most of these studies were based on face-to-face peer programs. Further exploring the RUBY participants’ views on different aspects of the support, how it was different from other support, and gaining a deeper understanding of women’s experience could aid the implementation of this intervention at a larger scale. Peer training could incorporate aspects of support that mothers found positive and helpful, ensuring a framework that facilitates engaging and effective delivery of support. In-depth qualitative analyses of participants’ experience of proactive telephone-based peer support interventions is limited. A recent RCT of proactive telephone peer support (in conjunction with face to face Lactation Consultant support) in the context of a low-middle income setting reported peer supporters provided ‘moral support’ and sharing their experiences helped the mother continue breastfeeding, however mothers perceived the support provided no benefits to their social network [29]. To fill this gap in the literature, this paper aims to provide an in-depth understanding of women’s experience of receiving proactive telephone-based peer support in a trial which was able to successfully increase breastfeeding maintenance at six-months.

## Methods

### Design

This qualitative study was conducted over a 12-month period (December 2015- November 2016), utilising face-to-face interviews. In this paper we draw on interviews with ten women to focus on an in-depth understanding of the experience of women who had received telephone-based peer support as part of the RUBY RCT. Full ethics approval was obtained from La Trobe University (HEC 12–082), Royal Women’s Hospital (HREC 12/25), Monash Medical Centre (HREC 12251B) and Western Health (HREC/12/WH/107).

### Recruitment

All 504 women allocated to the RUBY peer support intervention who had completed a 6-month postpartum telephone interview were mailed a survey exploring their experiences of peer support. In December 2015 an option was added to this survey for women to indicate their willingness to be contacted to take part in a one-to-one interview. Names and contact details of women agreeing to be contacted were passed to researcher, McLardie-Hore (FMc), who telephoned all women to discuss and offer participation in a face-to-face interview. The researcher (FMc) met with all women who agreed to participate in their homes and obtained written informed consent, prior to interview. We aimed to continue interviewing until data collection yielded no further themes i.e., data saturation had been reached. While in reality the number of women willing to participate dictated when recruitment ceased, by the last few interviews, repetition of concepts was noted, suggesting that data saturation had been reached.

### Data collection

A semi‐structured interview schedule was developed for the study and was made up of four open-ended questions exploring the woman’s experience of receiving volunteer peer support, what the relationship was like, what (if any) impact the support had, and if the support differed from other supports she may have received, with a final question asking if there was anything else the woman would like to talk about. The interview schedule was based on concepts of social support theories in particular three theoretical perspectives described by Lakey and Cohen [30]. The first is stress and coping, in which social support provides some protective effects against stress. Secondly, a social constructionist perspective which views social support as influencing health through promotion of self-regulation and self-esteem. The final is the relationship perspective, which asserts support provides the concurrent positive effects of companionship, intimacy, and low social conflict. Each of these was used to help inform the interview questions. Author FMc conducted all the interviews face-to-face with participants, with interview duration between 20–65 min. Interviews were audio recorded, and transcribed verbatim by FMc.

### Data analysis

Analysis of participant interviews was conducted using the thematic networks analytic method developed by Attride-Stirling [31]. This method of qualitative thematic analysis was chosen to provide insight to the shared meanings and experiences of participants [32]. The interview transcripts were ‘reduced’ into manageable, but meaningful segments producing basic (or sub) themes. Further exploration of these basic themes allowed researchers to create linkages between the ‘explicit statements and the implicit meanings’ [33] in participants’ conversations. These linkages melded the basic themes into unified organising themes representing the common subject matter derived from the discourse. In order to further explore and explain the principal meanings of these accounts, the organising themes were read and re-read, checking back to the text and basic themes, then organised into the unifying global theme. Transcription and first coding of each interview was conducted by FMc. Another member of the research team (HMc) read all the interviews, cross-checked the codes for validity and consistency of interpretation, and then the emerging themes were discussed between all members of the team until consensus was reached. This analysis was approached reflexively to put aside researchers’ prior knowledge, recognise the potential impact of the researchers’ own perspectives, and allow a true representation of participants’ experiences [34]. Vignettes from participant interviews are used to illustrate the basic and organising themes, and subsequent global theme, with participant descriptors used for each quote [Number participant “#_” age in years “_yrs,”, length of peer support in months “PS_ mths”, and duration of breastfeeding in months “BF_”].

## Results

### Participants

Of the 100 mailout surveys sent with the option for further contact for interview, a total of 63 were returned, out of which 34 women declined, five did not respond regarding participating in the interview (effectively declining), and 24 women agreed to be contacted. Of those 24, 10 women consented to participate, 11 were unable to be contacted, two declined participation at that point, and one had moved interstate. Women were offered options as to where to be interviewed, but all chose their own homes, which were located in inner city and metropolitan Melbourne, and regional Victoria. At the time participants were recruited to the RCT (between 11 and 15 months previous, a day or two after their baby was born), most felt their partners and family/friends were either supportive of their feeding choices or preferred them to breastfeed, and most felt they had a lot of support from family and friends for breastfeeding. At the time of interview, participants were aged between 26 and 37 years, and most lived with their partner (Table 1). Participants’ duration of breastfeeding ranged from 5 to 13 months, including two who were continuing to breastfeed. Women received the peer support for a median period of 5.5 months, ranging from four to six months. Eight participants in this study were born in Australia.

**Table 1:** Participant characteristics

*At time of recruitment to RUBY trial*								*At time of interview*		
**#**	**Country of birth**	**Age**	**Partnered**	**Partner view of feeding**	**Family view of feeding**	**Family & friends support for breastfeeding**	**Intended duration of breastfeeding**	**Duration of peer support**	**Duration of breastfeeding**	**Baby age**
1	Australia	36	No partner	NA^a^	Prefers BF	Moderate	6 mths or longer	6 mths	13 mths ongoing	13 mths
2	Australia	37	Living with partner	Supportive of my choice of feeding^a^	Supportive of my choice of feeding^a^	Little	6 mths	5 mths	5 mths	14 mths
3	Australia	28	Married	Supportive of my choice of feeding^a^	Supportive of my choice of feeding^a^	A lot	6 mths or longer	4 mths	11 mths ongoing	11 mths
4	Australia	32	Living with partner	Supportive of my choice of feeding	Don't know what they think	Moderate	6 mths or longer	5 mths	13 months	13 mths
5	Australia	27	Married	Prefers BF	Supportive of my choice of feeding^a^	A lot	6 mths or longer	6 mths	12 mths	14 mths
6	Australia	33	Living with partner	Supportive of my choice of feeding^a^	Supportive of my choice of feeding^a^	A lot	10 mths	5 mths	11 mths	14 mths
7	Vietnam	33	Married	Prefers BF	Supportive of my choice of feeding^a^	A lot	No plans for how long	6 mths	7 mths	11 mths
8	Australia	32	Married	Supportive of my choice of feeding^a^	Doesn’t mind	A lot	No plans for how long	6 mths	7 mths	15 mths
9	Bangladesh	33	Married	Prefers BF	Prefers BF	A lot	18 mths	6 mths	7 mths	13 mths
10	Australia	26	Living with partner	Supportive of my choice of feeding^a^	Supportive of my choice of feeding^a^	Moderate	6 mths	6 mths	11 mths	11 mths

*NA* Not applicable, *mths* months ^a^no matter which I do

### Themes

Five themes representing common subject matters were derived from the interview transcripts to form an overarching global theme of non-judgemental support and guidance.

### Non-judgemental support and guidance

**Fig. 1:**
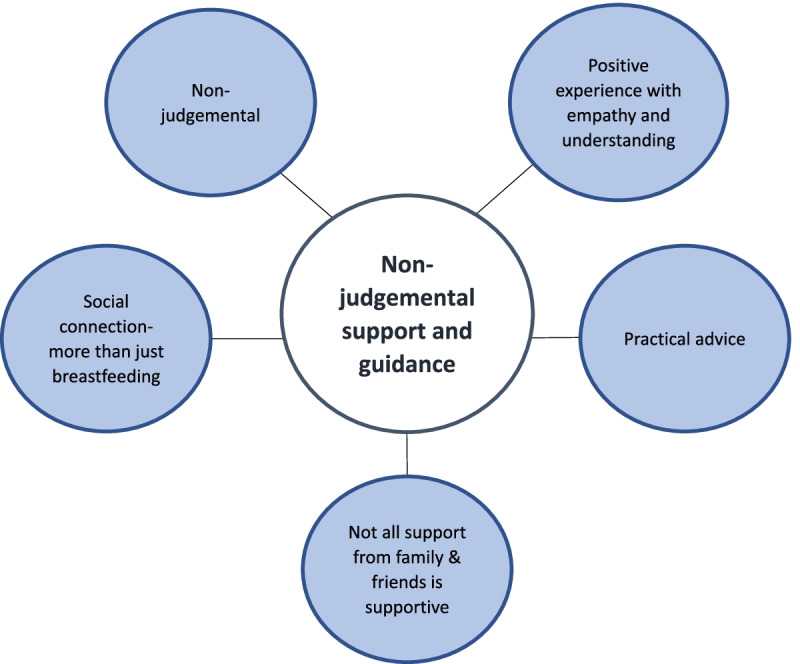
Global theme and organising themes

The overall global theme of ‘non-judgemental support and guidance’ included five organising themes, four of which reflected women’s positive experiences of the peer support. These themes were ‘non-judgemental’, ‘a positive experience with empathy and understanding’, ‘practical advice’ and ‘a social connection-more than just breastfeeding. The remaining theme contrasted the support received from others, this theme was ‘not all support from family and friends is supportive’ (Fig. 1). Each of the organising themes are discussed below.

### Non-judgemental

*She always made it clear that it was just her like trying to give me some ideas to try, it wasn’t like she was trying to push things onto me or um you know…I didn’t feel like she was judging me, I think that’s what the biggest part was.* (#10 36yrs, PS 6mths, BF 6mths)

Unlike supportive interactions with family, friends and health professionals, women described feeling relieved that they could be ‘upfront and honest’ with their peer and that the support was unconditional. A number of women commented that the peer being ‘anonymous’ (having never met them) aided their ability to be open and frank.

*As well as perhaps someone who wasn’t perhaps so well connected to you know family wise, or you could be quite upfront and honest about what you’re experiencing is they wouldn’t… you want someone neutral…* (#6 33yrs, PS 5mths, BF 11mths)

*I think that the fact that they don’t know you, so that family and friends have set expectations potentially about how you would behave or what they’d assume your values are or a whole range of things…or just want the best for you, knowing you. Whereas I think someone who is really neutral … doesn’t know the background and is happy just to see you at surface value…I think there are so many emotions flying around, someone who can take the emotion out of the equation … is really helpful…so I think it is different kind of advice, like they’re not, you know there’s nothing in it that she has to please me. She’s not my friend. She doesn’t have to see me next week, you know. She can have that; I guess she can challenge that status quo if it needed to be.* (#6 33yrs, PS 5mths, BF 11mths)

*I mean it was different because umm…you know you’re talking to someone who you really only know their name… someone that’s experienced it on some level I think is very important…And um being anonymous as well I think was really important.”* (#4 32yrs, PS 5mths, BF 13mths)

Many mothers talked about the pressure and judgement from health professionals, which they compared with the support from their peer. In interactions with health professionals some women felt they couldn’t ask questions for fear of judgement and were often left feeling uncertain and doubting their abilities as a new mother. Many of the interactions left women feeling stressed and disempowered.

*… and there’s a lot of pressure there, is your baby actually getting enough and having a really hard time with the maternal health nurses whether they mean to or not mean to, that feeling really grilled, about their choices, and that you know potentially whether they should have gone to formula or they should be doing something else.* (#6 33yrs, PS 5mths, BF 11mths)

*…I guess if you’re feeling uncertain about something you…often you don’t want to talk to someone who is a professional because you don’t want to be seen as ahh unknowing.* (#4 32yrs, PS 5mths, BF 13mths)

### Positive experience, with empathy and understanding

A strong and consistent theme for the participants in the study was that they found the peer support to be a positive experience.

*… she absolutely relayed some of that sort of unconditional support going you know ‘You’re doing a great job.” It was really positive, very positive, almost like a coach, cheerleader, kind of role.* (#6 33yrs, PS 5mths, BF 11mths)

*…for me it was a really positive experience…those calls and contacts through my first few months… really gave me a lot of confidence to …keep going and I’m grateful that I was able to do it actually. …it really did give me confidence and I’m still going because of that support I had…”* (#3 28yrs, PS 4mths, BF ongoing)

Peers were described as encouraging, reassuring empowering, and someone the mother could relate to. Peers bolstered women’s confidence with a number of women attributing this support to their continuation of breastfeeding.

*… But yeah, she was, she was really positive for me and I think if it wasn’t for her …… having that reassurance I actually don’t think I would have continued breastfeeding*.” (#3 28yrs, PS 4mths, BF ongoing)

*… a really big impact for me …like say I’m a first time Mum, and umm also the pressures of breastfeeding, it’s so demanding just having that support, reassurance, from someone other than…that you can relate to, another mother.* (#3 28yrs, PS 4mths, BF ongoing)

*So, hearing someone else’s story from their similar, opposite but similar experience… it really helps to, helps you understand that it’s, don’t stress too much about it, and you know your baby better than anyone else. And she, did very well at that I thought she was very reassuring. (*#8 32yrs, PS 6mths, BF 7mths)

*… They’re just there to really, I guess understand what you’re going through and be there to help encourage you to persist with it, or go through challenges or you know, keep going.* (#6 33yrs, PS 5mths, BF 11mths)

*…when I was in my sleep deprived state, just reassuring me, that you know, everyone goes through it, umm, I guess stages when I was really down, umm, she would sort of… reassure me that everything’s normal, and a lot of people go through this and it’s a stage with the baby and everything... (*#3 28yrs, PS 4mths, BF ongoing)

Participants described the empathy and understanding from their peers. Through empathetic listening, and sharing their own experiences, peers were able to relate to, validate and normalise what the mothers were going through.

*… it was nice to have somebody who was, you know. Who was engaged and who was just listening to what I said, and understood…* (#2 37yrs, PS 5mths, BF 5mths)

*… another opinion and that could maybe talk through…when you’re trying to seek out answers or you’re trying to understand that your experience is normal* (#5, 27yrs, PS 6mths, BF 11mths)

*…I think she was really good and just said ‘Look, you know it’s completely up to you, have confidence in your decisions* (#6 33yrs, PS 5mths, BF 11mths)

### Practical advice

Participants described a broad range of practical advice offered by their peer, from breast milk supply, expressing and storage of breast milk, management of sore breasts, nipple pain, and nursing bras, as well as advice on baby sleep, massage and health.

*… I had some lumps in my breasts…and they were really, really sore…um and I had an idea that they were… some sort of blockage…she did offer some very good advice for getting in the shower and…So it never progressed any further than that…* (#8 32yrs, PS 6mths, BF 7mths)

*So that kind of ticked something new over in my brain, so yeah, those casual anecdotal conversations I think, hmmm and just suggestions and broad advice, or places to look*. (*#*1 36yrs, PS 6mths, BF ongoing)

Women appreciated that peers provided unbiased options, talking through issues, clarifying questions and concerns, reducing the mother’s uncertainty and confusion. Peers guided mothers to reliable sources of information or when needed, to seek further help from community supports or health professionals.

*I know I’ve been in situations where I haven’t had a good headspace, and having that person that can absolutely direct you to different sources of information or help run through it when it seems confusing. (*#6 33yrs, PS 5mths, BF 11mths)

*I was so stressed because I couldn’t express and don’t know how to express…and she helped me and to relax me…and all the information.* (#7 33yrs, PS 6mths, BF 7mths)

### A social connection—more than just breastfeeding

Women described other benefits that the peer support provided, with the interactions being more than just breastfeeding including the peer ‘checking in’ on the mother and baby’s health.

*…she would always remember things or had noted down things that I’d ask and that was always she’d check in* (#1 36yrs, PS 6mths, BF ongoing)

*…it wasn’t like, we would talk mostly about breastfeeding, but we also talked about other bits and pieces. So, she would ask how [baby] was sleeping and try and help in that way and that was really nice, …you know and the other mothers in my mothers’ group were dealing with their own disasters, so you know, umm so it was nice to have that support, as well.* (#2 37yrs, PS 5mths, BF 5mths)

The peer provided women with a social connection, even for those women who hadn’t felt socially isolated, and this was valued by the mothers. They described the peer as friendly and warm, a ‘normal person’ on the other end of the phone. The relationship with the peer was described as informal, but often close, and women appreciated another mother who celebrated their progress and milestones, shared their own personal experiences and helped the mother feel ‘grounded’. They viewed the support by telephone as simple, and the proactive nature made it easy and accessible.

*Which I feel like …someone who is close to you and gives you all experience that you need, and yeah.* (#7 33yrs, PS 6mths, BF 7mths)

*… in that time, even though you’re surrounded by people you still feel… quite alone. …and I think that our little stories, and she’s always had something funny in return, so I when I had my child and some ‘poo-nami happened’… little stories like that…Makes you feel like you’re not so alone.* (#8 32yrs, PS 6mths, BF 7mths)

*… it was almost like we were friends. The first conversation pretty much really warm and umm it was someone I was really …say I looked up to I didn’t ever meet her face to face. But someone, I really valued what she said, so I really valued our …I would call it a friendship I would think.* (#3 28yrs, PS 4mths, BF ongoing)

### Not all support from family and friends is supportive

In this final theme, women talked about the support from family and friends, how this support did not always meet their needs and was often a negative experience. They described ‘well-meaning’ family and friends who had their own agendas, different views or weren’t open to talking about the needs of the mother. Family and friends would tell the mother how, and what to do and often left her doubting herself and her abilities to parent. This support was contrasted with that from the peer, in which their ‘neutrality’ was viewed as a positive attribute and the support was focused on the mother and her wellbeing.

*Of course, they [family & friends] are there to support you, but I think having someone there, another Mum, who is not your family or in your circle, not your social circle, just there purely for support for breastfeeding made a huge difference…even when I was doubting myself, hearing things from what I would call my support network, my friends and family, I would always relate back to her.* (#3 28yrs, PS 4mths, BF ongoing)

*…the pressures of breastfeeding, it’s so demanding just having that support and reassurance, from someone other than…that you can relate too, another mother. Umm because it’s all well and good and well having the support of your partner and everything else…you have the support of family and friends [but] not everyone in your family network either breastfed, agrees…do you know what I mean?...they don’t all have the same values.* (#3 28yrs, PS 4mths, BF ongoing)

*I think just having someone who you could have that honest conversation with that was happy to listen to it…without trying to impose their [views, like family and friends might] …oh well you know ‘I found it easier…and don’t you think the benefits outweigh it’ and they’d be justifying their own decisions…*(#6 33yrs, PS 5mths, BF 11mths)

*With all the friends, like the mother[s] group, we the same age…we compare this is my baby, that is my baby…we seem like talk the problem…more than try to solve the problem [laughs]* (#7 33yrs, PS 6mths, BF 7mths)

## Discussion

This paper provides an insight into the experiences of first-time mothers receiving proactive telephone-based peer support in the context of a RCT aimed at increasing breastfeeding, in Victoria, Australia. Consistent with, and strengthening the findings reported in our paper on women’s views from the RUBY study [21], mothers described their experience as positive, reassuring, with the peer normalising their experiences and the non-judgemental, empathetic support helping them to cope with the many challenges faced.

Our themes and global theme are similar to a number of other studies, and support theoretical perspectives described by Lakey and Cohen [30]. The global theme is consistent with other studies that have reported the importance of non-judgemental, empathetic support [35, 36]. The social connection with their peers, is described by mothers as helping them to feel less alone [27] and isolated [37], as a friendship, someone close [38] and at times a confidante. This connection better enabled participants to be relaxed, frank and open in their conversations, with the relationship providing positive effects from companionship and intimacy [30]. The peer was described as non-judgemental [35, 39] empathetic and understanding, attributes which allowed for low-social conflict within the relationship. Participants felt they were able to better cope and manage the stressors [40] of breastfeeding and parenting, through the peer sharing their own stories, normalising the mother’s experience [28, 40, 41] and helping them to understand their struggles were not unique, supporting the mother’s self-regulation. With the encouragement and reassurance from the peer boosting self-esteem and helping mothers continue breastfeeding [29, 42].

Interview questions in this study were informed by social support theories, and whilst responses can be viewed through this social support lens, how this support, provided during the transition to motherhood, assists women should not be regarded in the same manner as support for physical or mental health conditions. The expectations of modern Western society are that women should be strong, confident, not needing help and are able to cope with many competing and conflicting demands [43]. While many new mothers may feel overwhelmed and uncertain, they may also view asking for help as a failure [44], and choose not to discuss negative feelings, fearing they will be judged and measured as a bad or inadequate mother. Feeling like they have failed in this new role can serve to threaten their sense of self and their identity [45]. Consequently, many women perpetuate, rather than resist, the ideology of motherhood as instinctive and natural, and that mothers ‘can do it all’. Viewing new motherhood in this manner, aids a better understanding of how women seek, receive and view support, both positively and negatively during this period. The peer’s non-judgemental, understanding and empathetic approaches to conversations gave mothers ‘permission’ to be open and honest, allowing them to drop any façade of ‘coping with it all’. The anonymity of the peer (they had never met, and only first name and telephone details were provided) may have added another layer of protection allowing for non-judgemental support, with women describing ‘not knowing her’ as helping them to be honest and open. Anonymous communication has been associated with individuals being more likely to seek support and disclose stressful experiences when dealing with a stigmatised illness [48]. Even within face-to-face breastfeeding peer support groups, Taylor (2019) describes women’s fear of judgement, ‘unhelpful advice and disparaging remarks’[46] In this way the telephone-based approach, as opposed to face-to-face, can be seen as not only easy and accessible, but also facilitating non-judgemental support through anonymity.

At the time of recruitment, most mothers in this study described having ‘a lot’ of support for breastfeeding from family and friends, and that their partner and family and friends were ‘supportive no matter how’ they fed their baby, however for many women, the way in which this support was enacted was very different to their expectations. Whilst advice and support from within the mothers’ ‘circle’ of family and friends may have been available and wanted, the manner in which this support was offered or given, as seen elsewhere, was not what the mothers needed, [27] and led to the mothers feeling judged, [41, 45] inadequate, and disempowered. [45–47] Women felt others had their own agendas and were not supportive of their needs, but rather imposed their own ideals, offering inappropriate advice, [39] and causing conflict. Similarly, as seen in other studies, women described feeling pressured [46] and appraised by health professionals [48], not being able to ask basic questions, or reveal their true feelings or issues, for fear of being seen as a bad or inadequate mother. Even when women were surrounded by others, some described feeling alone. The new mother’s fear of judgement and the resulting lack of effective support from family, friends and health professionals was frequently countered by the support of the peer.

Whilst this study supports others findings that women value support via telephone, [23] one qualitative study in the context of a low-middle income setting[29] reported mothers would have preferred face-to-face contact with their peer, describing support via telephone as a barrier to social connection. It is, therefore, important to ensure that there is not a ‘one size fits all’ approach to providing peer support for breastfeeding mothers, and to consider the nuances of differing cultural needs and the context of the individual’s life.

## Conclusion

Telephone-based peer support for breastfeeding was highly valued by women in this study. Women appreciated the non-judgemental, empathetic and understanding contact from peers. This approach, facilitated by the anonymity of the telephone-based program, allowed more open and honest conversations, normalising their experience and helped women feel less alone in their challenges with breastfeeding and in the transition to motherhood. These findings, in the context of a high-income country, can inform the design of innovative, sustainable models of peer support, delivering effective and engaging support with reach to a broad population.

## Data Availability

The datasets used and analysed during the current study are available from the corresponding author on reasonable request.
